# Frontal EEG-Based Multi-Level Attention States Recognition Using Dynamical Complexity and Extreme Gradient Boosting

**DOI:** 10.3389/fnhum.2021.673955

**Published:** 2021-06-01

**Authors:** Wang Wan, Xingran Cui, Zhilin Gao, Zhongze Gu

**Affiliations:** ^1^State Key Laboratory of Bioelectronics, School of Biological Science & Medical Engineering, Southeast University, Nanjing, China; ^2^Key Laboratory of Child Development and Learning Science, Ministry of Education, School of Biological Science & Medical Engineering, Southeast University, Nanjing, China; ^3^Institute of Biomedical Devices (Suzhou), Southeast University, Suzhou, China

**Keywords:** attention recognition, sustained attention task, electroencephalogram, dynamical complexity, extreme gradient boosting

## Abstract

Measuring and identifying the specific level of sustained attention during continuous tasks is essential in many applications, especially for avoiding the terrible consequences caused by reduced attention of people with special tasks. To this end, we recorded EEG signals from 42 subjects during the performance of a sustained attention task and obtained resting state and three levels of attentional states using the calibrated response time. EEG-based dynamical complexity features and Extreme Gradient Boosting (XGBoost) classifier were proposed as the classification model, Complexity-XGBoost, to distinguish multi-level attention states with improved accuracy. The maximum average accuracy of Complexity-XGBoost were 81.39 ± 1.47% for four attention levels, 80.42 ± 0.84% for three attention levels, and 95.36 ± 2.31% for two attention levels in 5-fold cross-validation. The proposed method is compared with other models of traditional EEG features and different classification algorithms, the results confirmed the effectiveness of the proposed method. We also found that the frontal EEG dynamical complexity measures were related to the changing process of response during sustained attention task. The proposed dynamical complexity approach could be helpful to recognize attention status during important tasks to improve safety and efficiency, and be useful for further brain-computer interaction research in clinical research or daily practice, such as the cognitive assessment or neural feedback treatment of individuals with attention deficit hyperactivity disorders, Alzheimer’s disease, and other diseases which affect the sustained attention function.

## Introduction

Sustained attention refers to the ability to focus on task-related information stimuli while consciously trying to ignore other stimuli over a relatively long period. As a foundational cognitive function, sustained attention underlies other cognitive domains, such as learning and memory (Fortenbaugh et al., 2017). However, due to the lack of a monitoring mechanism, assessing whether people are sustainably focused and improving concentration in learning activities is a challenge. Many studies have applied electroencephalography (EEG) to explore neural mechanisms because it has both high time-resolution and applicability (Schu, 1999). In addition to being used to diagnose various brain-related diseases, EEG has shown great potential in studying related brain activities such as cognition, memory, and emotion. It is also an essential measurement for assessing attention status (Aoki et al., 1999; Müller et al., 2000; Palva and Palva, 2007; Srinivasan et al., 2009).

It has been shown that EEG activities in different frequency bands can be related to specific physiological states. The traditional EEG analysis method is to divide the brain activity into different frequency bands, including delta (1–4 Hz), theta (4–8 Hz), alpha (8–13 Hz), beta (13–30 Hz), and gamma (30–60 Hz) waves (Teplan, 2002). A lot of studies were considering band power as an important parameter to characterize the state of attention. EEG power-based indices [P_β_/P_α_, 1/P_α_, and P_β_/(P_α_ + P_θ_)] were used to assess the sustained attention level in healthy controls and diffused axonal injury patients, and they found significant negative correlations between P_β_/P_α_, 1/P_α_ indices and the variations of mean reaction time during sustained attention test (Coelli et al., 2018). Hu et al. (2018) extracted the Hjorth parameters and power spectral features to distinguish three attention levels evaluated by a self-assessment model according to 10 subjects’ self-reports during a learning process. They proposed a combined procedure with correlation-based feature selection and k-nearest neighbors classification algorithm to achieve the highest accuracy of 80.04%.

In addition to linear characteristics, nonlinear analysis methods have great potential in EEG analysis based on its nonlinear and nonstationary characteristics. Nonlinear dynamical analysis make it possible to study self-organization and pattern formation in the complex neuronal networks of the brain (Stam, 2005). Several studies have reported the association between attentional function and EEG single-scale complexity. Bob et al. (2011) using pointwise correlation dimension to analyze attentional processes related to dissociative states. Rezaeezadeh et al. (2020) focused on entropy measures, including univariate features from individual EEG channels and multivariate features from brain lobes, to diagnosis Attention Deficit Hyperactivity Disorder. Recent studies have shown that applying nonlinear multiscale information analysis to EEG can provide new information about the complex dynamics of brain cognitive function, such as emotion recognition (Gao et al., 2019). Ke et al. (2014) conducted two experiments instructing all subjects to perform tasks with three different levels of attention (i.e., attention, no attention, and rest). Nonlinear parameters including entropy and multiscale entropy were extracted, and a Support Vector Machine (SVM) model was performed for classification between each experiment state and resting states, with 76.19 and 85.24% accuracy, respectively, in the two experiments.

The relationship between EEG activity and attention state is not limited to EEG amplitude changes with experimental tasks, it also includes phase and cross-frequency coupling. Hanslmayr et al. (2005) calculated the phase-locking index using Gabor wavelet analysis with a frequency resolution of 0.5 Hz during a continuous visual target stimulus processing task. The results suggested that focused attention will cause a large phase locking of alpha wave without amplitude change. Szczepanski et al. (2014) found increases in power of high gamma (70–250 Hz) in electrocorticography (ECOG) signal during allocation of visuospatial attention, and these high gamma power increases were modulated by the phase of the ongoing delta/theta (2–5 Hz) phase. Borhani et al. (2019) applied brain network connectivity analysis based on Granger causality on event-related selective attention tasks, and found that the flow of information between independent neural components on the left occipital cortex and the right supplementary motor area became highly coupled on alpha waves during the selective attention tasks. The dynamical analysis is an extension of the EEG measures with a focus on the time sequence of EEG index or functional connectivity networks (Bola and Sabel, 2015; Pagnotta et al., 2020). Previous studies based on perfusion functional magnetic resonance imaging found that the connectivity strength of frontal-parietal network represented by topological characteristics dynamically changes to compensate the cognitive decline during long-term sustained attention task (Taya et al., 2018). The dynamical analysis represents the evolution of neural activity, mostly used is dynamic functional connectivity. At present, the correlation between dynamical sequence of EEG complexity measures and sustained attention performance in the aspect of helping assess attention during sustained attention tasks still need research proof.

To date, little research has been done on the identification of multiple attention levels using continuous EEG feature analysis instead of superposition of event related potentials. Previous studies mostly allowed subjects to control or self-assess their attention level by subjective methods (Li et al., 2013; Chen et al., 2017). Due to the reliable of continuous attention test, and the neural mechanism of continuous attention has been widely recognized (Rosvold et al., 1956), we performed an AX-continuous performance test (AX-CPT) task to get EEG segments of different attention levels. The entropy and multiscale entropy based dynamical complexity analysis were performed for discriminating attention states in four levels. The quantified complexity indices were used as a feature vector to classify different attention levels through Extreme Gradient Boosting (XGBoost). Furthermore, the relationship between EEG complexity indices and task response performance was studied to assess the effectiveness of dynamical complexity analysis in attention recognition. Our hypotheses for this study were that (1) during sustained attention tasks, the reaction time becomes faster or slower is corresponded to different attention state, and this change of attention level can be reflected in the variation of EEG dynamical complexity; (2) the multi-scale nonlinear method will provide more information than the single-scale complexity in recognition of different attention states, and (3) the EEG dynamical complexity-based attention recognition may be more sensitive and effective in frontal region than other brain regions.

## Materials and Methods

### Experiments

#### Participants

This study included 42 right-handed subjects (16 males, 26 females) with age ranging from 20 to 26 years (mean age 24.26 ± 1.17). All participants had no history of neurological or psychological disorder, and they all had normal vision or were corrected to normal vision (self-report). This study was approved by IEC for clinical research of Zhongda Hospital, affiliated to Southeast University (No. 2019ZDSYLL073-P01). All participants signed the informed written consent form before participating in the experiment.

#### Sustained Attention Task

In this study, the attention continuous performance test (CPT) task was employed to assess the sustained attention capability of the participants, while their continuous EEG signals were recorded.

**FIGURE 1:**
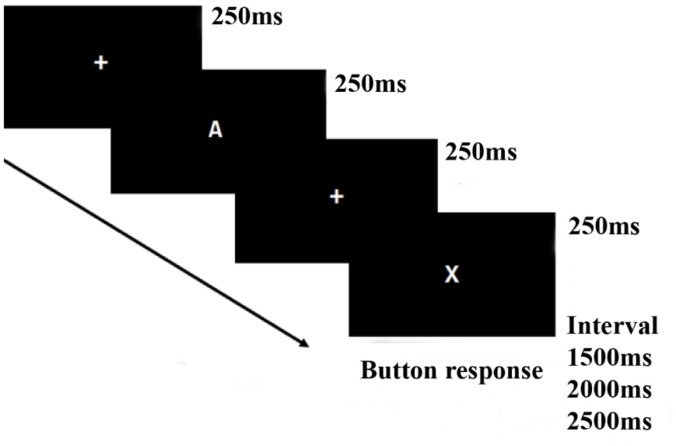
AX-Continuous performance test (AX-CPT) task flow.

The version of the CPT task used in this study was the AX-CPT (Cohen et al., 1999). During the AX-CPT task (see Figure 1), the participants were presented with a series of letters of English alphabet randomly, in which they were instructed to inhibit their response when the target sequence is “X” preceded by an “A” and to make a response as fast as possible for other target sequences different from sequence “AX.” There were 192 trials, with 144 “AX” sequences (Left mouse button response) and 48 other sequences (right mouse button response), each sequence contained two letters. Each letter stimulus was presented for 250 ms with an inter-stimulus interval (ISI) of 250 ms and an inter-trial interval (ITI) of 1,500, 2,000, or 2,500 ms randomly set. The task was performed in a dimly lit and quiet room.

#### Questionnaires

All questionnaires were administered to participants after EEG recording, including demographic (age, gender, and handedness), Pittsburgh Sleep Quality Index (PSQI) questionnaire (Buysse et al., 1989), Profile of Mood States (POMS) questionnaire (Curran et al., 1995), and Cognitive Failures Questionnaires (CFQ) (Broadbent et al., 1982).

### EEG Recording and Preprocessing

The EEG data were recorded by 32 electrode cap (Easycap) based on the international 10-10 system (Fp1, Fp2, Af3, Af4, F7, F3, Fz, F4, F8, Fc5, Fc1, Fc2, Fc6, T7, C3, Cz, C4, T8, Cp5, Cp1, CP2, Cp6, P7, P3, Pz, P4, P8, Po3, Po4, O1, Oz, O2) and digitized at 1,000 Hz using the Neuroscan Synamp2. The reference electrode is located near Cz. Eye movements were recorded using two bipolar electrodes (one electrode superior to the right eye, another electrode to the right of the orbital fossa). The impedance of each electrode was below 10 kΩ.

A notch filter at 50 Hz to suppress the remained power-line noise, and a band-pass filter at 0.3–70 Hz using a FIR filter with a 3rd order Butterworth window was used to eliminate movement artifacts. Independent Component Analysis (ICA) was performed for removing EEG ocular artifacts (Vigário, 1997). The 32-channels EEG was decomposed into 30 independent components (ICs) by ICA, and then the Electro-oculogram (EOG) were automatically recognized by calculating the correlation between each IC and two EOG signals. The noise ICs were set to zero, and the other ICs were reconstructed to EEG without ocular noise. The clean EEG was segmented into 3 s sections according to each AX-CPT trial for feature extraction.

**FIGURE 2:**
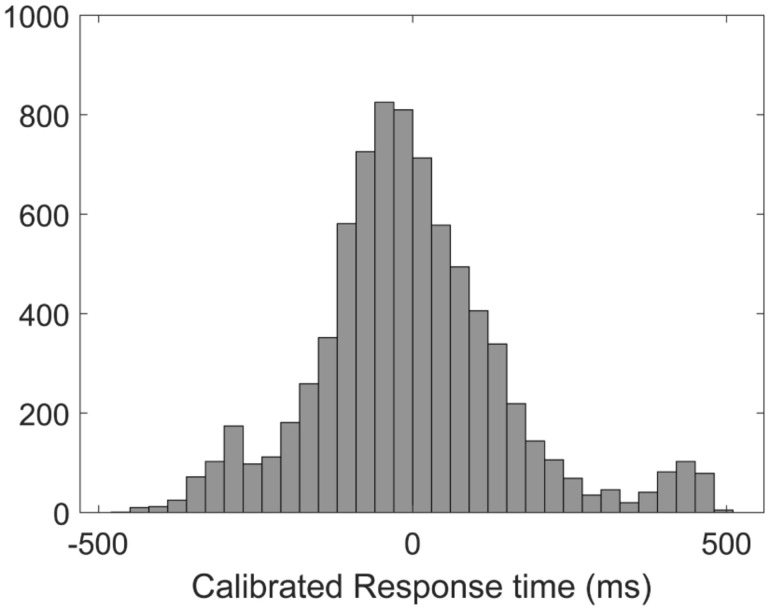
The distribution of the calibrated response times (C-RT).

Previous studies have shown that response time may be an indicator of attention level (Gunawan et al., 2017). When people are sustaining a high level of concentration, they usually respond to visual stimuli immediately and quickly. Conversely, people with low attention levels usually make slower response. In fact, the ability to respond to tasks rapidly varies with different individuals, so it would happen that some subjects respond relatively slowly on all trials, and some subjects respond relatively quickly on all trials. In this case, evaluation and definition of different attention levels directly by absolute reaction time in a cross-subject attention recognition will not be accurate enough. Hence, for the purpose of cross-subject attention states recognition, we corrected the task response times based on the average reaction time of each person to obtain calibrated response times (C-RTs). Since the AX-CPT task is composed of 192 trials and each trial corresponds to a calibrated response time, the EEG data is segmented according to the event of the task trial with a window length of 3 s. After removing the outliers of C-RTs, an average of 190.76 (±2.55) task epochs segments (1 epoch = 3 s) for each subject were remained. As we can see in Figure 2, the distribution of C-RTs in the histogram is very similar to the log-normal distribution.

In this context, we set a critical value α based on the C-RTs to match EEG segments with different attention states. we defined task data epochs with C-RTs significantly shorter than the critical value as “high attention state,” data epochs with C-RTs significantly longer than the critical value as “low attention state,” and other data segments with medium C-RTs were considered as “medium attention state.” For example, α = 0.25 defined that the data segments with C-RTs in the top 25% (shorter response times) represented “high attention state,” the data segments with C-RTs in the bottom 25% (longer response times) represented “low attention state,” and other segments represented “medium attention state.” To increase the reliability of this definition, different significance level α (0.05–0.35) were picked up to represent different attention levels in Results section. Besides the task states, we also took the 3-min resting state EEG of each subject for analysis and obtained 60 resting data epochs (1 epoch = 3 s) for each subject.

### Frequency Domain Features

Wavelet Packet Decomposition (WPD) (Coifman and Wickerhauser, 1992) projects the time series onto the space of orthogonal wavelet basis functions and decomposes the signal into low frequency and high frequency. Compared with wavelet analysis, it not only decomposes the low-frequency part of the signal, but also re-decomposes the high-frequency part. In this paper, 7-layer WPD was used to obtain theta (θ, 4–8 Hz), alpha (α, 8–13 Hz), and beta (β, 13–30 Hz) band oscillations. The power ratio features β/θ, β/α, β/(α+θ) calculated by Welch’s power spectral density estimate methods (Welch, 1967) were seen as classical EEG features for attention recognition in this study.

### Single-Scale Complexity

#### Approximate Entropy

Approximate entropy (ApEn) (Pincus, 1991) uses a non-negative number to represent the complexity of a time series and reflect the occurrence of new information in the time series. For a given time series [*u*(1),*u*(2),…,*u*(*N*)], two parameters *m* and *r* are defined to compute ApEn. First, take *m* consecutive points in sequence to form vectors sequence

(1) U⁢(i)={u⁢(i),u⁢(i+1),…,u⁢(i+m-1)}

where *i* = 1,2,…,*N*−*m* + 1 then define the distance between the two vectors *U*(*i*) and *U*(*j*) as

(2) $$d\,\left[U\,\left(i\right),U\,\left(j\right)\right]=\underset{k=0,1,2,..,m-1}{m\,a\,x}\left|u\,\left(i+k\right)-u\,\left(j+k\right)\right|$$

where *i* = 1,2,…,*N*−*m* + 1. For a given threshold *r*, count the number of *d*[*U*(*i*),*U*(*j*)] < *r* as *N*_*m*_(*i*), and the ratio of *N*_*m*_(*i*) to the total number of distances *N-m+1* is recorded as $C_{m}^{r}\,\left(i\right)$,

(3) $$C_{m}^{r}\,\left(i\right)=N_{m}\,\left(i\right)/\left(N-m+1\right)$$

Take the logarithm of $C_{m}^{r}\,\left(i\right)$ and calculate its average values for all *i*, denoted as ϕ_*m*_(*r*),

(4) $$\phi_{m}\,\left(r\right)={\left(N-m+1\right)}^{-1}\,\sum_{i=1}^{N-m+1}l\,n\,C_{m}^{r}\,\left(i\right)$$

Increase the dimension to *m +* 1, and recalculate ϕ_*m* + 1_(*r*) according to the above steps, the approximate entropy of the sequence is

(5) $$A\,p\,E\,n\,\left(m,r,N\right)=\phi_{m}\,\left(r\right)-\phi_{m+1}\,\left(r\right)$$

The parameters for ApEn was set as *m* = 2,*r* = 0.2.

#### Sample Entropy

Sample entropy (SampEn) is an improved complexity measurement based on the concept of ApEn (Richman and Moorman, 2000). The initial calculation process is the same as ApEn, but there is an additional restriction that *i*≠*j* for the calculation of *d*[*U*(*i*),*U*(*j*)] in formula (2).

SampEn is calculated as

(6) $$S\,a\,m\,p\,E\,n\,\left(m,r,N\right)=\ln\left(\phi_{m}\,\left(r\right)/\phi_{m+1}\,\left(r\right)\right)$$

The parameter for SampEn was set as *m* = 2,*r* = 0.15.

#### Fuzzy Entropy

Fuzzy entropy (FE) uses a fuzzy membership function to measure the degree of similarity of vectors (Chen et al., 2007), so the calculated values are smooth, continuous, and more robust. The phase-space reconstruction is performed on time series [*u*(1),*u*(2),…,*u*(*N*)], and a set of *m*-dimensional (*m* ≤ *N-2*) vectors are reconstructed as follows:

(7) $$X_{i}^{m}=\left\{u\,\left(1\right),u\,\left(2\right),\ldots ,u\,\left(i+m-1\right)\right\}-u_{m}\,\left(i\right),i=1,2,\ldots ,N-m+1$$

where *u*_*m*_(*i*) represents the mean of this *m*-dimensional vector.

The maximum Euclidean distance between $X_{i}^{m}$ and $X_{j}^{m}$ was defined as $d_{i\,j}^{m}$, given *n* and *r*, the degree of similarity of two vectors can be calculated according to the fuzzy membership function:

(8) $$A_{i\,j}^{m}=u\,\left(d_{i\,j}^{m},n,r\right)=\exp\left(-{\left(d_{i\,j}^{m}\right)}^{n}/r\right)$$

Define the function φ^*m*^(*n*,*r*) as

(9) $$\varphi^{m}\,\left(n,r\right)=\frac{1}{N-m}\,\sum_{i=1}^{N-m}\left[\frac{1}{N-m-1}\,\sum_{j=1,j\neq i}^{N-m}A_{i\,j}^{m}\right]$$

The fuzzy entropy for the given time series can be defined as

(10) $$F\,u\,z\,z\,y\,E\,n\,\left(m,r,n,N\right)=l\,n\,\varphi^{m}\,\left(n,r\right)-l\,n\,\varphi^{m+1}\,\left(n,r\right)$$

The parameters for FuzzyEn was set as *m* = 2, *r* = 0.15, *n* = 2.

### Multiscale Complexity

#### Multiscale Sample Entropy

Multiscale Entropy analysis is a method to investigate the complexity of time series at multiple time scales (Costa et al., 2005). Multiscale sample entropy (MSE) extends sample entropy to multiple time scales or resolutions. The basic principle of MSE involves coarse-graining and entropy calculation procedure. For a given time series[*u*(1),*u*(2),…,*u*(*N*)], the range of scale factor τ is defined from 1 to *s*, and the original time series was coarse-grained according to the scale factor τ as follows:

(11) $$y_{j}^{\tau }=\frac{1}{\tau }\,\sum_{i=\left(j-1\right)\,\tau +1}^{j\,\tau }u\,\left(i\right),j=1,2,\ldots ,i\,n\,t\,\left(N/\tau \right)$$

Calculate the SampEn for each coarse-grained series $\left\{y_{j}^{\tau }\right\}$ in different scale factors.

(12) $$M\,S\,E\,\left(\tau ,m,r\right)=S\,a\,m\,p\,E\,n_{y_{j}^{\tau }}\,\left(m,r,N\right),\tau =1,2,\ldots ,s$$

#### Multiscale Fuzzy Entropy

The calculation principle of multiscale fuzzy entropy (MFE) (Zheng et al., 2014) are similar to the steps of MSE. After the coarse-graining procedure in all scale factors, MFE is defined as:

(13) $$M\,F\,E\,\left(\tau ,m,r\right)=F\,u\,z\,z\,y\,E\,n_{y_{j}^{\tau }}\,\left(m,r,N\right),\tau =1,2,\ldots ,s$$

When analyzing multiscale complexity, we used *m* = 2, *r* = 0.15 for MSE and *m* = 2, *r* = 0.15, *n* = 2 for MFE, and scales were set from 1 to 50.

### Extreme Gradient Boosting

Extreme gradient boosting (XGBoost) model was used as a classifier to distinguish different levels of sustained attention. XGBoost is an efficient and distributed implementation of the Gradient Boosting algorithm (Chen and Guestrin, 2016). The main advantage of XGBoost lies in its scalability, which allows parallel and distributed computing, and makes learning and model exploration faster. And approximate algorithm used in XGBoost finds the candidate set of cutting points according to the quantile of the feature distribution, and then traverses all the sub-sets to determine the best split point. This method replaces the greedy algorithm that needs to traverse all samples when looking for the best segmentation point in training. In addition, XGBoost proposed a more regularized model formalization to prevent over-fitting, so that the performance of XGBoost is better than the conventional Boosting algorithm. Apart from that, XGBoost is an unexplored algorithm in the field of attention recognition. So here in this work we explored this algorithm to get better accuracy. The subject leave-one-out cross-validation (LOOCV) and 5-fold-cross validation were both performed to evaluate the model’s predictive performance. XGBoost library through the python package was used to complete this work.

### Statistical Analysis

The D’Agostino-Pearson normality test was performed to check whether the data satisfied the normal distribution. The parametric ANOVA test was used for the data that satisfied normality. If the data are not normally distributed, a non-parametric Kruskal-Wallis test was used.

To uncover the associations between the dynamical analyzed indices and behavioral parameters, Spearman’s correlation coefficient was performed between the EEG features and calibrated response time (C-RTs). The significant difference was defined as the *p*-value < 0.05. All statistical analysis methods were performed in MATLAB R2018a.

## Results

### Behavior Analysis

Descriptive statistics were presented in Table 1, behavioral results were reported in terms of mean reaction time (RT) (ms) and mean errors (%) for all subjects. The average PSQI score (mean = 5.31, *SD* = 2.09) can be used as evidence to support that the currently selected group of subjects does not have insomnia disorder (Dietch et al., 2016). The average Total Mood Disturbance (TMD) score of POMS (mean = 108.31, *SD* = 20.60) shown that the emotional state of participants is normal and there is no negative mood swing [TMD score range from 68 to 268, a higher TMD score is indicative of greater mood disturbance (McNair et al., 1992)]. The average CFQ score (mean = 52.90, *SD* = 12.47) suggested that the concentration of participants in daily life is at a normal range (Wallace et al., 2002). No abnormal value was found in the PSQI, POMS, and CFQ questionnaires.

**TABLE 1:** Mean task results of all subjects.

Characteristics	Mean (standard deviation)	n	%
**Subject**			
Male		16	38.10
Female		26	61.90
**Age (years)**	24.26 (1.17)		
Education level	college student	42	100
Handedness	right-handed	42	100
**AX-CPT task**			
Mean RT (ms)	405.04 (96.52)		
Mean errors (%)	8 (6.65)		
**Questionnaires**			
PSQI	5.31 (2.09)		
POMS	108.31 (20.60)		
CFQ	52.90 (12.47)		

### EEG Dynamical Complexity Between Resting and Attention States

**FIGURE 3:**
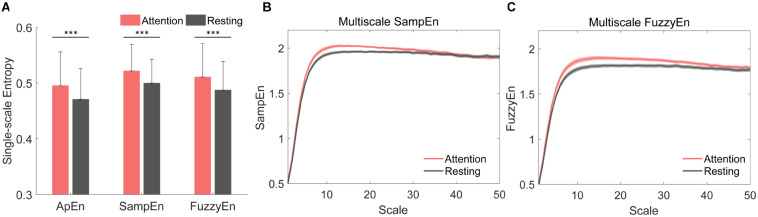
Comparison of complexity measured separately by approximate entropy (ApEn), sample entropy (SampEn), fuzzy entropy (FuzzyEn), Multiscale sample entropy (MSE), and Multiscale fuzzy entropy (MFE) between resting state and sustained attention state at Fz. **(A)** The averaged values of single-scale complexity (ApEn, SampEn, and FuzzyEn) for 42 subjects [^∗∗∗^*p* < 0.001, the error bar represented standard deviation (SD)]. **(B)** The averaged Multiscale complexity measured by MSE and **(C)** MFE for 42 subjects [shadows represented standard error (SE)].

We analyzed the ApEn, SampEn, FuzzyEn, MSE, and MFE of all 32 channels for all EEG epochs to investigate the single-scale and multiscale complexity, and the group difference of EEG complexity between resting state (eyes opened) and sustained attention state was presented in Figure 3. The three single-scale complexity values during the sustained attention task were significantly higher than resting state (Figure 3A, ApEn, *p* < 0.001, SampEn, *p* < 0.001, and FuzzyEn, *p* < 0.001). Distinct increases of multiscale complexity curves (scales 1–50) measured by MSE and MFE were observed during sustained attention state (Figures 3B,C). These promising results suggest that complexity has the potential to distinguish different levels of attention states.

### EEG Dynamical Complexity Compared Among Four Attention Levels

#### Single-Scale EEG Complexity

The single-scale EEG complexity for different attention levels was explored using three kinds of entropy calculation methods, i.e., ApEn, SampEn, and FuzzyEn. Four different levels of attention states were defined in this study, including high attention (HA), medium attention (MA), low attention (LA), and resting state (RS). The definition criterion for different attention levels was introduced in section “EEG Recording and Preprocessing.” Here, we first took α = 0.25 as the representative case in this section, and finally α = 0.05–0.35 were evaluated accordingly in section “Effects of Significance Level Alpha.”

**FIGURE 4:**
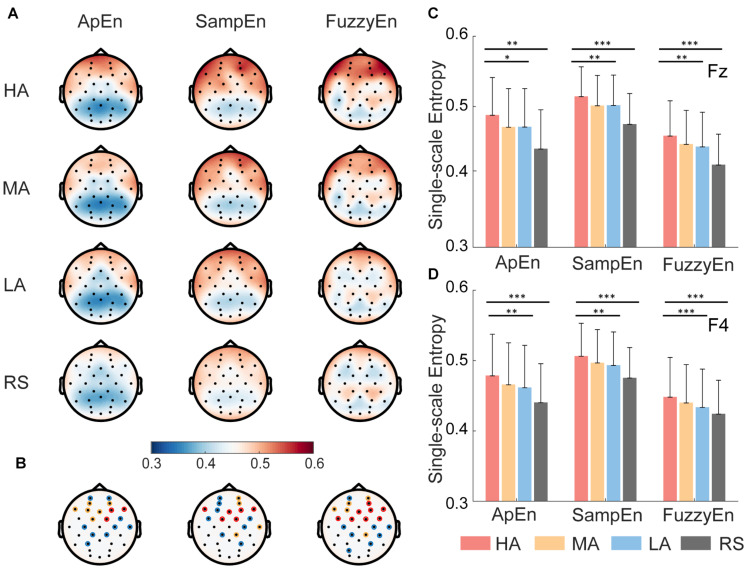
Brain topography and representative results of single-scale EEG complexity. **(A)** Topography of approximate entropy (ApEn), sample entropy (SampEn), and fuzzy entropy (FuzzyEn) for all 42 subjects at four attention states (i.e., high attention—HA, medium attention—MA, low attention—LA, and resting state—RS). The red color means high entropy, and blue means low entropy. **(B)** The statistical significance of 32 channels using Kruskal-wallis test. The color of circle indicates significance level α, blue represents α in the range of (0.01, 0.05], yellow represents α in the range of (0.001, 0.01], red represents α smaller than 0.001. **(C,D)** Representative results of single-scale complexity measured group differences for three attention levels (HA, MA, and LA) and four different attention levels (HA, MA, LA, and RS) in frontal region at Fz and F4. Nonparametric Kruskal-Wallis test, ^∗^*p* < 0.05, ^∗∗^*p* < 0.01, ^∗∗∗^*p* < 0.001. Error bars = ±1 SD.

The brain topography of averaged ApEn, SampEn, and FuzzyEn for all 42 subjects was shown in Figure 4, representing HA, MA, LA, and RS, separately. These topography maps demonstrate that (i) consistently in all three entropy methods, higher attention levels showed higher single-scale EEG complexity than lower attention levels in frontal and central brain regions, whereas resting state showed lowest EEG complexity than keeping focused (see Figure 4A), and (ii) these differences among the four attention levels in frontal and central regions were highly significant (evaluated by nonparametric Kruskal-Wallis test, see Figure 4B).

Furthermore, as illustrated in Figures 4C,D, we took Fz and F4 as representative results to show the group differences in frontal regions. FuzzyEn achieved highest statistical difference than ApEn and SampEn among HA, MA, and LA states, representatively shown in Figures 4C,D, ApEn (Fz: *p* < 0.05, F4: *p* < 0.01), SampEn (Fz: *p* < 0.01, F4: *p* < 0.01), and FuzzyEn (Fz: *p* < 0.01, F4: *p* < 0.01). Approximately equal significant group differences were found among four attention levels (*p* < 0.001 for both SampEn and FuzzyEn measures in both Fz and F4). Similar group differences were found in other EEG channels (i.e., channels marked with the red asterisk in Figure 4B).

Thus, the comparison results of single-scale complexity measured by ApEn, SampEn, and FuzzyEn showed that most of the frontal lobe electrodes (e.g., FP1, FP2, AF3, AF4, F7, F3, Fz, F4, F8, Fc1, Fc2) and part of the parietal lobe electrodes (e.g., C3, C4) all have significant differences among different attention levels, suggesting that characterization of different attention levels is more associated with EEG complexity in the frontal and central regions than other brain regions.

#### Multiscale EEG Complexity

**FIGURE 5:**
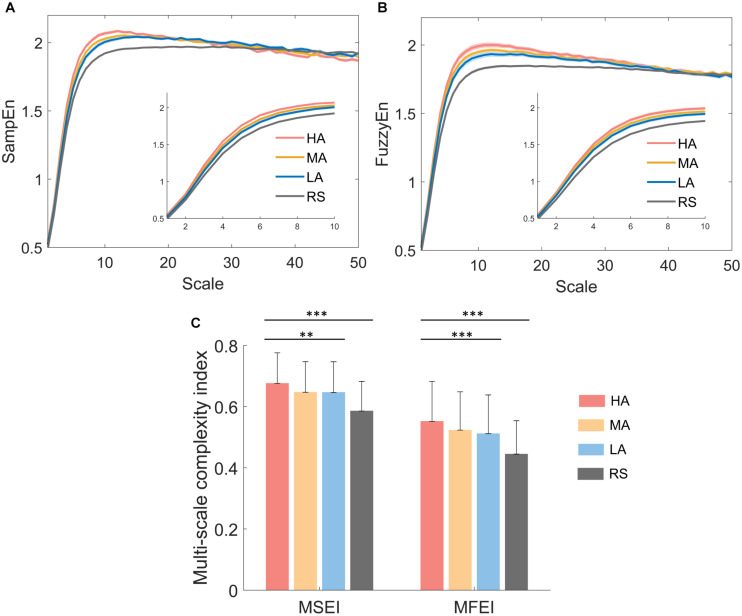
Representative results of multiscale complexity analysis on electrodes F4. **(A)** The averaged multiscale sample entropy curves and **(B)** averaged multiscale fuzzy entropy curves of four attention states for 42 subjects, and the shadow represents SE. **(C)** Group differences between MSEI and MFEI among different attention levels. ^∗^*p* < 0.05, ^∗∗^*p* < 0.01, ^∗∗∗^*p* < 0.001. Error bars = SD.

The dynamical complexity measured from the temporal regularity of EEG oscillations across different time scales may provide more information. The multiscale EEG complexity of three different attention levels and resting state were analyzed by multiscale measures of both MSE and MFE. The averaged MSE and MFE curves of F4 were shown in Figure 5. As in the enlarged detail plots at scales 1–10, more obvious difference was found in MFE curves (Figure 5B) than MSE curves (Figure 5C) among four attention states, showing that the higher the attention level is, the higher the entropy value at this scale. In Figure 5A, the mean sample entropy values of MA and LA are approximately equal across different time scales. At time scales 11–50, the curves of four attention levels are aliased together in both Figures 5A,B. Hence, the area under MSE and MFE curves at time scales 1–10 was defined as multiscale sample entropy index (MSEI) and multiscale fuzzy entropy index (MFEI). Figure 5C displayed the group differences among both three different attention levels (i.e., HA, MA and LA, MSEI: *p* < 0.01 and MFEI: *p* < 0.001) and four attention levels (i.e., HA, MA, LA, and RS, MSEI: *p* < 0.001 and MFEI: *p* < 0.001).

### Recognition of Multi-Level Attention

#### Classification Results

For the dynamical complexity method, both single-scale (i.e., ApEn, SampEn, FuzzyEn) and multiscale complexity indices (i.e., MSEI, MFEI) extracted from EEG recordings in the frontal region (i.e., FP1, FP2, AF3, AF4, F7, F3, Fz, F4, F8, FC5, FC1, FC2, FC6) were fed into the classification model. To prove the efficiency and advantage of complexity-based features, the conventionally used power ratios of different frequency bands which have been widely considered to be related with attention level (Barry et al., 2009; Putman et al., 2010), e.g., β/θ, β/α, β/(α+θ), were applied as classical method to recognize different level of attention states. For the classical method, 3 power ratio features β/θ, β/α, β/(α+θ) was extracted from the same frontal electrodes. Hence, the feature dimension of each sample in the complexity-based model is 65 (5^∗^13) and for the classical model, each sample is described by 39 (3^∗^13) features. To eliminate the influence of individuality on classification, the features of all data segments were separately normalized to 0–1 in all subjects, respectively, before classification.

Furthermore, in order to further evaluate the distinguish performance of the Complexity-XGBoost, SVM, and random forest (RF) were also performed, which have been commonly used in EEG analysis. The parameters optimizing process was performed for each classifier model during training.

Table 2 demonstrated the classification results of multi-level attention recognition obtained from both the LOOCV and 5-fold cross-validation. With LOOCV, the proposed Complexity-XGBoost model achieved accuracy of 64.69, 70.49, and 76.39%, respectively for four-level attention states classification (HA, MA, LA, and RS), three-level attention states classification (HA, MA, and LA), and two-level attention states classification (attention state (AS), and RS). With 5-fold cross-validation, the Complexity-XGBoost model achieved accuracy of 81.39, 80.42, and 95.36%, respectively.

**TABLE 2:** The performance between XGBoost and other machine learning methods in the LOOCV and 5-fold cross-validation.

Methods	Model	LOOCV			5-Fold cross validation		
			
		4-Level	3-Level	2-Level	4-Level	3-Level	2-Level
Classical methods	SVM	59.23 ± 8.32	65.14 ± 7.79	73.82 ± 8.72	62.42 ± 0.51	72.00 ± 1.73	82.32 ± 0.81
	RF	60.19 ± 5.21	64.12 ± 4.96	72.34 ± 7.46	63.52 ± 0.65	73.39 ± 1.10	81.71 ± 1.37
	XGBoost	63.15 ± 4.63	65.24 ± 3.86	73.39 ± 4.47	67.45 ± 0.48	72.75 ± 1.03	83.46 ± 1.83
Complexity analysis	SVM	60.13 ± 12.32	70.34 ± 9.76	75.43 ± 13.45	78.46 ± 1.12	78.25 ± 1.19	94.34 ± 1.18
	RF	63.77 ± 8.63	72.33 ± 10.22	73.70 ± 11.68	77.27 ± 1.73	77.68 ± 0.90	94.12 ± 0.34
	XGBoost	64.69 ± 6.20	70.49 ± 4.59	76.30 ± 9.24	81.39 ± 1.47	80.42 ± 0.84	95.36 ± 2.31

Compared with classical methods, complexity-based methods resulted in best performance in all two validations and three classification strategies, and this advantage is more distinct when using the 5-fold cross-validation. When both LOOCV and 5-fold cross-validation was conducted, the performance in XGBoost outperformed those of other two machine learning methods. The results of the SVM were not much different from those of the RF.

**FIGURE 6:**
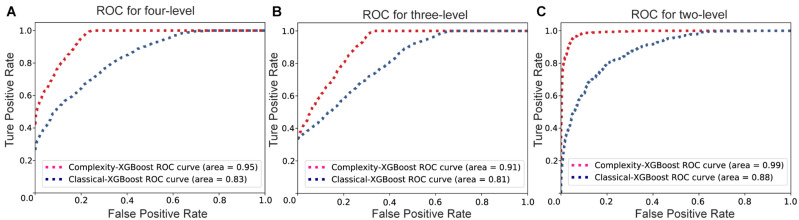
The Receiver Operating Characteristic (ROC) curves of Complexity-XGBoost and Classical-XGBoost methods for three classification strategies. **(A)** The ROC curve for four-level classification. **(B)** The ROC curve for three-level classification. **(C)** The ROC curve for two-level classification.

The Receiver Operating Characteristic (ROC) curves of Complexity-XGBoost and Classical-XGBoost methods for three classification strategies are showed in Figure 6. The area under curve (AUC) of Complexity-XGBoost method are 0.95, 0.91, and 0.99 respectively, for four-level, three level, and two-level attention states classification. And the AUC of Classical-XGBoost method are 0.83, 0.81, and 0.88, respectively, for three kinds pf classification strategies.

#### Feature Importance

**FIGURE 7:**
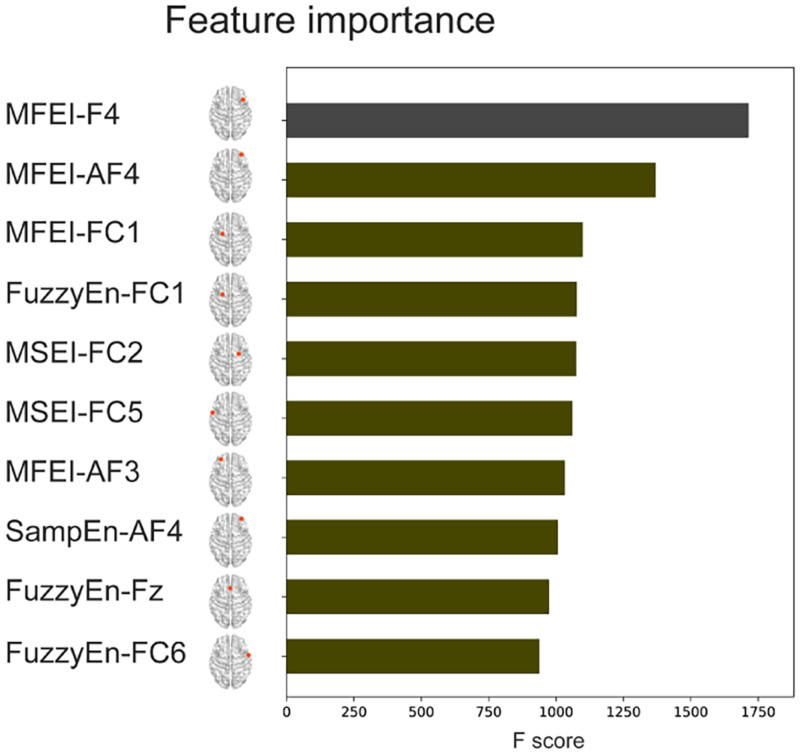
Feature importance of Combined-XGBoost (top 10 features).

The complexity-based features and the classical PSD features from 32 channels were combined to conduct a Combined-XGBoost classifier. One advantage of this approach is that we can retrieve the importance score of each feature after constructing the gradient boosted trees to obtain the importance ranking of the feature. The top 10 important features and their electrode positions are shown in Figure 7. The top 10 important features were all complexity-based features. MFEI appears 4 times in the top 10 important features, FuzzyEn 3 times, MSEI twice, and SampEn once, indicating that the complexity-based features in identifying different attention levels were far better than the PSD features. Meanwhile, the electrode positions where these features are located also showed that the frontal lobe brain area is of great significance for identifying different level of attention.

#### Effects of Significance Level Alpha

**FIGURE 8:**
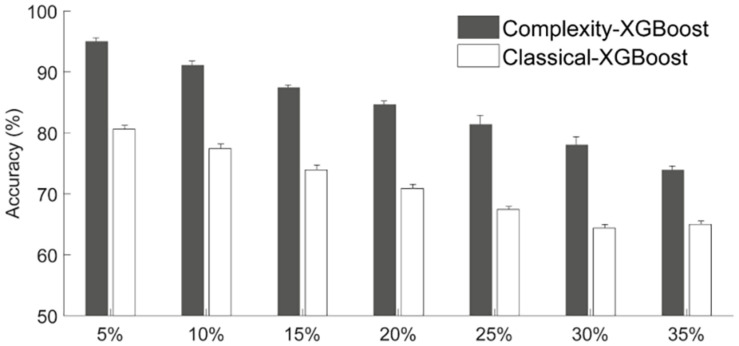
The effects of the significance level α to classification accuracy.

The above results were obtained based on the definition criterion α = 0.25 for different attention levels (introduced in sections “EEG Dynamical Complexity Compared Among Four Attention Levels” and “Recognition of Multi-Level Attention”). Here, to investigate the reliability of complexity-based features for characterizing specific attention level, we evaluated the multi-level attention recognition accuracy under different significance level α = 0.05–0.35 (see Figure 8, 4-level attention classification). With the increase of α-level, the classification accuracy of classical methods based on the power ratio tends to decrease dramatically from 80.59% (α = 5%) to 64.40% (α = 30%), while the methods based on complexity analysis have much higher accuracies (73.91–95.01%) for the recognition of the four attention states. These findings further confirmed the advantages of nonlinear complexity analysis for attention-related EEG recognition. Hence, in this study we took α = 0.25 as the representative threshold to define HA, MA, and LA is reliable.

### Relationship Between EEG Dynamical Features and Real-Time Response During Sustained Attention Task

Brain dynamics research has highlighted the contributions of the ongoing EEG to behavioral responses. In this section, we examined the correlation effects of state-related EEG changes on stimulus-response efforts during sustained attention task. First, the averaged time-varying C-RTs across subjects were calculated. As for multi-channels EEG features, we performed principal component analysis (PCA) method to realize dimensionality reduction. PCA and related techniques have been applied to describe the fluctuation of EEG measurements during the resting state (Leonardi et al., 2013), continuous movie-watching task (Demirtaş et al., 2019), and whole-brain connectivity dynamics (Allen et al., 2014; Zhu et al., 2020). PCA is a method accepted by many researches to reduce the dimensionality of multi-channel or whole brain features, and then to study dynamic fluctuation. PCA was performed on the dynamical EEG features of 13 electrodes in the frontal brain area within each subject, then the first principal component (PC1) was selected as the representative EEG features for each subject. Finally, the averaged time-varying PC1 of 42 subjects were calculated. These correlation effects were estimated using a Spearman’s correlation test between averaged EEG features and averaged C-RTs of all subjects along with data segments over time.

Five complexity-based features (ApEn, SampEn, FuzzyEn, MSEI, and MFEI) and 3 power ratio features [β/θ, β/α, and β/(α+θ)] are all took into consideration separately. Significant negative correlations were found between the involved EEG features (except the classical features β/α) and C-RTs, MFEI had the highest correlation (*r* = −0.35, *p* < 0.001).

## Discussion and Conclusion

This study proposed to use a calibrated response times (C-RTs) to obtain multi-level attention states during an AX-CPT sustained attention test, which can truly reflect the changes in attention without the influence of individuality on response. The proposed entropy-based Complexity-XGBoost model achieved outstanding performance, respectively, in recognizing four, three, and two levels of attention states relative to a similar model trained on conventional power spectral based measures. Furthermore, we found significant correlation relationships between complexity-based EEG features and C-RTs over time.

EEG signal is a nonlinear coupling of large number of nerve cells. The linear EEG analysis can evaluate the communication between neural networks in the same oscillating frequency band or similar neuron firing patterns. However, it is not clear how much information is missing since the behavior of neural network can be highly nonlinear and nonstationary (Yang et al., 2018; He and Yang, 2021). Thus, nonlinear analysis methods like entropy and complexity are more suitable for EEG feature extraction than the power spectral based linear analysis. The dynamical complexity of the neural network should correlate with the conscious state of the subject (Tononi and Edelman, 1998). The neural network based on automatic behavior or low-control behavior should have lower dynamical complexity than the neural network that consciously controls behavior, such as controlling oneself to maintain a high attention state.

Compared with the performance of previous studies on the EEG application of attention state monitoring (Chen et al., 2017; Hu et al., 2018; Gaume et al., 2019; Jeong and Jeong, 2020), we dealt with the recognition up to four levels of attention states and our performance is higher than them using Complexity-XGBoost (see Table 3). The Complexity-XGBoost achieved the accuracy of 81.39 ± 1.47% for four-level attention states (HA, MA, LA, and RS), 80.42 ± 0.84% for three-level attention states (HA, MA, and LA), and 95.36 ± 2.31% for two-level attention states (AS and RS) when using 5-fold cross-validation. With LOOCV, the accuracies were 64.69 ± 6.20%, 70.49 ± 4.59%, and 76.30 ± 9.24%, respectively. The performance of the three-level attention classification (without RS) is still relatively high, indicating that the accuracy of the four-level classification is not affected by the obvious difference between resting state and attention state.

**TABLE 3:** Comparison with previous studies on attention recognition.

Authors	Attention task (levels)	Subjects	Methods	Window (seconds)	Brain regions (channels)	Validation	Accuracy (%)			
							**2-Levels**			
	
Chen et al. (2017)	Continuous performance task (high-attention, low-attention)	10	Temporal and entropy features—SVM	Trial length	Prefrontal (1)	3/4 train, 1/4 test	91.60			

							**3-Levels**			
Hu et al. (2018)	Randomly selected learning task (high, neutral, low)	10	Linear and nonlinear features—CFS+KNN	180	Central and temporal (6)	10 times 3-Fold CV	80.84			

							**2-Levels**		**3-Levels**	
	
Gaume et al. (2019)	Continuous performance task (easy, medium, and hard)	14	Power features—LDA	5	Whole brain (16)	Leave-one-subject-out	75		51.8	
				30			85		64.8	

							**2-Levels (in-ear)**		**2-Levels (prefrontal)**	
	
Jeong and Jeong (2020)	Psychomotor vigilance tasks (attention, rest)	6	Temporal and spectral features—Echo State Network	0.5	In-ear (2) prefrontal (2)	Within-subject	81.16		82.44	
						Cross-subject	64.00		65.70	
						10-Fold CV	74.15		73.73	

							**2-Levels**	**3-Levels**		**4-Levels**
	
Our study	AX-CPT (rest, LA, MA, HA)	42	Complexity—XGBoost	3	Frontal (13)	Leave-one-subject-out	76.30	70.49		64.69
						5-Fold CV	95.36	80.42		81.39

**SVM, support vector machine; CFS, correlation-based feature selection; KNN, k-nearest-neighbor; LDA, Linear discriminant analysis; ESN, Echo State Network; CV, cross-validation.**

The proposed attention recognition model based on the XGBoost algorithm adds a regularization step to the traditional gradient enhancement algorithm, which can reduce the degree of overfitting of training and improve the performance of cross-subject classification. Moreover, the Complexity-XGBoost model supports multi-threaded parallel computing, and the approximate algorithm is used to replace the greedy algorithm when looking for the best segmentation point, thereby greatly improving the computing efficiency of the algorithm and reducing the computational cost in real-time training. In terms of the recognition of small and medium-sized data, such as attention recognition using EEG features, algorithms based on XGBoost are by far one of the best ways for an application purpose. So attention recognition based on Complexity-XGBoost model has great application potential in actual portable brain-computer interface applications.

Furthermore, the interpretable property of the XGBoost algorithm also showed the importance of complexity-based features and frontal brain region. The presented attention recognition results in this study were obtained using EEG features derived from frontal brain region instead of the whole brain. The frontal region is involved in the generation of top-down control signals for attention transition, especially the prefrontal lobe area plays a vital role in the ability to switch attention control based on changing task requirements (Rossi et al., 2009). It is suggested that the frontal region played an important role in attention regulation (Daffner et al., 2000; Paneri and Gregoriou, 2017). Both the brain topography of three single-scale complexity indices (see Figure 4) and feature importance ranking with electrode locations (see Figure 7) showed that frontal channels could distinguish different levels of attention states more significantly than any other brain area. The frontal cortex, the control center for most cognitive functions, is considered a higher order area that controls several executive functions including taking charge of the brain’s attention and controlling relevant parts of the visual cortex (Baldauf and Desimone, 2014). However, previous studies found that other brain regions, including parietal and occipital, also reflect attentional modulation (van Schouwenburg et al., 2017; Magosso et al., 2019; Misselhorn et al., 2019). These studies focused on the relationship of attentional modulation and alpha oscillation or alpha power, which all used linear analysis methods. It appears that Hu et al. (2018) excluded frontal electrodes (they used C3, C4, Cz, P3, P4, Pz), whereas the this study focused on frontal electrodes. The mechanism that the linear analysis method and the nonlinear analysis method behave differently in different brain regions is worthy of further investigation. In this study, we proposed and verified the effective dynamical complexity indices for attention evaluation based on frontal EEG, which is a good impetus for the application of portable prefrontal EEG devices to promote real-time attention state assessment. Attention recognition based on Complexity-XGBoost could applied to the neural feedback treatment of diseases that affect cognitive function such as attention deficit hyperactivity disorder, mild cognitive impairment, or Alzheimer’s disease.

This study also provides an instance of EEG dynamical correlation analysis to investigate the effectiveness of EEG features in attention assessment. Our results showed that dynamical complexity measures were related to the changing process of response, e.g., dynamical complexity measure MFEI (*r* = −0.35, *p* < 0.001) were significantly correlated to the task performance during sustained attention task. Previous studies also demonstrated that attention dynamically modulates brain rhythms (Liu et al., 2020; McCusker et al., 2020; Pagnotta et al., 2020). McCusker et al. (2020) observed multi-spectral oscillatory robust effected by attention dynamically for both the directed and divided attention experiments in a MEG study. Liu et al. (2020) applied an complex analysis framework composed of weighted phase lag index and tensor component analysis and they found dynamic organizations of frequency-specific function connectivity can track the decrement and motivation of attention in sustained task. In a simplified perspective, dynamical complexity analysis conducted in this study may offer additional predictive value for attention.

One limitation of this study is that the time course of AX-CPT tasks was not sufficiently long, resulting in the reaction time not being able to progress overtime to produce more obvious changes. In future research, multi-session variable-speed AX-CPT tasks and longer experimental time will be performed to verify the advantages of nonlinear complexity methods for attention recognition. In addition, we also hope to use cross-frequency neural coupling measurement for attention recognition.

The present study investigated brain dynamical complexity concurrently during rest and a task characterized by sustained attention. The present findings demonstrated that dynamical complexity and XGBoost achieved great performance for different levels of attention states recognition, and significant differences were observed in the frontal regions.

## Data Availability Statement

The datasets for this article are not publicly available. Requests to access the datasets should be directed to XC, cuixr@seu.edu.cn.

## Ethics Statement

This study was approved by the IEC for clinical research of Zhongda Hospital, affiliated to the Southeast University (No. 2019ZDSYLL073-P01). The participants provided their written informed consent to participate in this study.

## Author Contributions

ZGu and XC conceived and designed the study. WW and ZGa performed the experiment and analyzed the data. WW drafted the manuscript. XC supervised the analysis, reviewed, and editing the manuscript. All authors contributed to the article and approved the submitted version.

## Conflict of Interest

The authors declare that the research was conducted in the absence of any commercial or financial relationships that could be construed as a potential conflict of interest.
